# Health beliefs and barriers related to HIV prevention and screening among students of the University of Vlora: a cross-sectional study

**DOI:** 10.1186/s12889-020-09416-8

**Published:** 2020-08-27

**Authors:** Rezarta Lalo, Gjergji Theodhosi, Alberta Breshanaj

**Affiliations:** 1 Faculty of Public Health, Department of Healthcare, University of Vlora “Ismail Qemali”, Skelë, Rruga Kosova, 9400 Vlorë, Albania; 2 Orthodox Clinic - Medical Center, Tirana, Albania; 3 University of Vlora “Ismail Qemali”, Vlorë, Albania

**Keywords:** Barriers, Health beliefs, HIV/AIDS, Screening, Sexual health, Student

## Abstract

**Background:**

Previous researchers have found that young university students can have a high level of knowledge about HIV/AIDS infection, but they are still not utilizing the existing HIV prevention methods. As a result there is a need to determine which factors and barriers influence the use of existing HIV screening and prevention methods among students of the University of Vlora in Albania.

**Methods:**

This was a cross-sectional study conducted among university students in the district of Vlora, Albania from April to June 2018. Stratified, multi-stage sampling technique was used to select randomly study subjects. A structured, self-administered questionnaire was used for data collection. Bivariate and multivariate logistic regression analysis was employed to reflect the relationship between variables.

**Results:**

The mean age of the participants (710) was 20.85 ± 2.1 years. 38% of them believe that chances of getting HIV would not stop them to have sexual intercourse with more than one partner, 69% report that using a condom seems like an insult to their partner. 78% of the students with sexual experience didn’t used Voluntary Counseling and Testing services. The students from the rural area (AOR = 0.50, 95% CI [0.30–0.82]) and those of first academic year (AOR second/first year =2.31, 95% CI [1.33–3.99], AOR third/first year =1.18, 95% CI [0.65–2.13]) were less likely to use HIV service.

**Conclusions:**

The findings reveal that health beliefs and barriers are good predictors of the preventive behaviours toward HIV infection. The survey has provided evidence to suggest that creating awareness about HIV prevention among student community, especially those from non-health sciences and rural areas could contribute to increased uptake of the VCT services, the condom use and to reduce the identified stigmatizing barriers.

## Background

There are many minor and major factors that help the HIV/AIDS (Human Immunodeficiency Virus/Acquired Immunodeficiency Syndrome) epidemic in national, regional, continental and global level. Even though the epidemics follow various courses in different countries, there are some common features as for the transmission and distribution method, which help us estimate the fast spreading of the virus globally [1]. The factors that raise the vulnerability to HIV include some cultural practices, the inappropriate control of health and resources, especially on health care, education and wellbeing, religious beliefs, poor governing, migration, conflicts, urbanizing, and stigma of the marginalized groups [2].

A significant diminuition in new cases of HIV infections has occured internationally, in specific 16% during these last 10 years, along with a reduction in number of deaths due to AIDS, 48% since 2005 [3]. Nonetheless, there are some countries around the world that have shown a different trend. In 2016, the majority of the European countries reported 160,453 new HIV cases in the World Health Organisation. This continued trend corresponds to a rate of 18,2 per 100,000 people [4]. While Albania remains a low HIV prevalence country, the rise of new HIV diagnoses in Albania is concerning. The European Centre for Disease Prevention and Control (ECDC) listed Albania third in the region in terms of affected individuals. According to ECDC, 94 albanian people had been affected by the HIV virus during 2017. Most of the cases are undergoing the developing phase of a disease, and the sum total of diagnosed people has gone to 1400. In Albania, as in the wider region, young people seem to be particularly vulnerable to HIV. The number of reported HIV- positive cases for youth aged 15–29 was about 22% of the total number of cases [4, 5].

A positive answer to the spread of HIV/AIDS starts with ***prevention*** [6]*.* Our country, Albania has followed the same line with the objectives of World Health Organization (WHO) and other scientific international institutions on HIV/AIDS prevention [7]. A lot of awareness campaigns are conducted in order to increase the youth awareness, so that they may be oriented toward a healthier life style, adopt positive behavior and increase the request for reproductive sexual services [8].

Various studies have reported that university students, who are mostly young people, rarely use existing HIV/AIDS preventive methods [9]. In Albania some studies report that the prevalence of not using condoms during the first sexual act is high in this age group [10] and the main reason is the pregnancy avoidance. Only a small percentage of the young people use the condom to avoid sexually transmitted diseases (STD) and HIV/AIDS [8]. In order to understand the behavior of young people who do not use condoms, it is essential to determine the beliefs and the perceived barriers that predispose condom use and non-use [11–13].

Another element which should be taken into consideration during the studies is the utilization of health services among the youth. Voluntary counseling and testing (VCT) is an effective approach for facilitating behavioral change, reducing unprotected sex around both preventing HIV as well as getting early access to care and suppor [14]. In Albania, VCT are services offered to all people who want to know their HIV status and a redesign of them is needed to better target and outreach to vulnerable populations. The data show that the use of health services from the youth is very low. Seeking an HIV test may be more difficult for young people because many lack experience and some barriers in accessing VCT services [15, 16]. The aim of the present study is therefore to evaluate health beliefs and barriers related to HIV prevention and screening with an emphasis on the association between VCT uptake and explanatory factors among students of the University of Vlora. To this end, this article addresses the following question: Will students’ beliefs influence health services uptake?

## Methods

### Study area, study design and study period

A cross-sectional study was conducted among university students, age ≥ 18 years, in the district of Vlora, Albania from April to June 2018. The study followed the consolidated criteria for observational research (STROBE).

### Sample size and sampling technique

Due to the lack of a similar study in this area, we used the Cochran formula to determine the sample size, which according to the calculations made, resulted in 384. As there is a total population of 8151 students in the university of Vlora, we made an adjustment [14] using the formula: nf = ni/[1 + (ni/N)], where ni is the sample size resulted by the Cochran formula and N is the sum total of all students in the University of Vlora. Using a 5% margin of error at a 95% confidence level, the required sample size was 367.

Multi-stage sampling technique was used to select study subjects. Firstly, the students were stratified by their field of study as health science and non health science. Secondly, two departments from health science and four departments from non health science were selected using simple random sampling based on proportion. Thirdly, the students in each field of study were further stratified by their year of study assuming that their field of study and duration of stay in the campus affect their VCT utilization. Finally, the students were selected from each batch proportionally by simple random sampling technique using computer generated random numbers.

### Data collection

Data was collected using an instrument in the form of a self-administered questionnaire of type Knowledge, Attitude, Practice and Belief survey (KAPB) which comprised four *sections.* The first section included the socio-demographic characteristics of participants, Section II presented students’ perceptions related to health beliefs and barriers of screening methods, Section III related to students’ knowledge about the preventive methods and Section IV on students’ sexual practices and VCT uptake. The perceptions related to Health Beliefs and Barriers were assessed using a ten items questionnaire and the evaluation was 1 score for the correct answer. Students could choose from 3 options for each question: “correct”, “incorrect” and “don’t know”. The “don’t know” answers were treated as incorrect answers in the analysis. An index of Health Beliefs and Barriers was created that ranged from 0 to 10, with higher scores indicating more correct perceptions. The perceptions’ classification was done in these levels: 6–10 points “correct” perception and 0–5 points “incorrect” perception. Knowledge on preventive methods was assessed by single question as “Using the condom during the sexual act may reduce the risk of HIV infection”. Sexual practices were assessed using three items of section III which includes sexual experience, the age of first sexual intercourse and number of sexual partners. VCT utilization was assessed by single question as “Did you used VCT service?” which respond in “Yes” or “No”.

The items were adapted from previous researches [14, 17–22] and developed for use in Albania. To increase the validity of the tool and the reliability of the statements, Section I to IV were based on various publically accessible survey instruments, such as the HIV-Knowledge Questionnaire [19], Attitudes about HIV Infection Questionnaire [20] adapted from a measure reported by Alawad, et al., AIDS Health Belief Scale (AHBS), developed by Zagumny and Brady [21], to measure the four components of the Health Belief Model (HBM), The Barriers to HIV testing scale-Karolinska version [22] reported by Wiklander et al.

The questionnaire is originally prepared in English language and then translated to Albanian and again retranslated to English by two language experts for consistency.

### Data analysis

An expert in statistic was used for data coding and analyses to enhance the research validity. For statistical analysis of the data was used statistical program SAS (Statistical Analysis System) version 9.1. For categorical variables were reported absolute numbers and percentages respective. To assess the association between categorical variables was used Chi-square statistical test. *P*-value ≤0.05 was taken as cut of value to be significant. A bivariate logistic regression analysis was used to reflect the relationship between the dependent variables (perception of health beliefs, VCT utilization) and the independent factors (socio-demographic, sexual behaviors). Bivariate analysis was also employed to assess the association between VCT utilization (dependent variable) and perceived barrier to HIV testing (independent variable); knowledge on preventive method (independent variable) and perceived barrier related to condom use (dependent variable). Variables with a bivariate association of *p*-value less than 0.05 were entered into the multivariate analysis. When we choose to analyse above variables using multivariate logistic regression, we also considered other criteria for its use: Dependent variable is measured in nominal scale, independent variables are nominal or continuous (number of sexual partners), observations are independent, lack of multicollinearity is controlled by calculating the Spearman correlation coefficient, by first coding the nominal variables. Adjusted odds ratios (AORs) and 95% confidence intervals (CIs) were presented along with the corresponding *p*-value in the multivariate analysis.

## Results

### Socio-demographic characteristics of participants

The mean age of the participants was 20.85 ± 2.1 years. Acoording to gender, about 56% of the participants were females and 44% males, related to residence, 66% of the students live in the city and 34% in the village. Related to civil status 83% are single, 7% are married, 9% live with another partner, 1% divorced. 68% of the participants have had sexual experience.

### The perception of health beliefs and barriers related to HIV/AIDS is shown in Table 1.

A total of 710 students completed the section on students’ perceptions of Health Beliefs and Barriers related to HIV/AIDS of the questionnaire. We found out that 69% of the students report that using a condom seems like an insult to their partner, 47% believe that a person infected with HIV can be identified from his/her thin look, 38% believe that chances of getting HIV would not stop them to have sexual intercourse with more than one partner and only 26% of participants would not get tested for HIV because of fear of losing partner.

**Table 1:** The evaluation of health beliefs & barriers related to HIV/AIDS among students of University of Vlora

The perception of health beliefs & barriers related to HIV/AIDS	Correct answer		Incorrect answer	
	No.	%	No.	%
1. I believe that a person should be worried about HIV/AIDS only if he/she starts to get sick.	476	67.04	234	32.96
2. I believe that a person infected with HIV can be identified from his / her thin look.	379	53.38	331	46.62
3. I believe that taking a test for HIV one week after having sex will tell a person if she or he has HIV.	486	68.45	224	31.55
4. I believe that HIV is a punishment for immoral behavior.	629	88.60	81	11.40
5. I believe that clients who get HIV through illegal behavior (e.g., sex work), should not be treated at hospitals and clinics.	442	62.25	268	37.75
6. I believe that religious people cannot get infected with HIV	558	78.69	152	21.41
7. The chances of getting HIV would not stop me to have sexual intercourse with more than one partner.	440	61.97	270	38.03
8. Using a condom seems like an insult to my partner.	218	30.70	492	69.30
9. I would not get tested for HIV because I was afraid of losing my partner.	520	73.34	188	26.66
10. I would not go to a local clinic to be tested for HIV because then everyone would know my status.	524	73.91	186	26.19

### The relationship between the perception of health beliefs & barriers and socio demographic variables is shown in Table 2.

Based on the scoring of perceptions’ classification, 76% of participants (540 students) had correct perception related to health beliefs & barriers. The results of *bivariate logistic regression analysis* for the relationship between the perception of health beliefs & barriers and socio demographic variables showed that there was a significant statistical association with variables “field of study” (Chi square *p* = 0.015 < 0.05) and “academic year” (*p* = 0.016). The results found that health science students were 1.6 times more likely than non health sciences ones to have correct perceptions (OR = 1.61; 95% CI, [1.12–2.32]). Furthermore, 82% of the students of the second year followed by those of the third year (78%) and the first year (68%) resulted to have correct perception of beliefs and barriers. According to bivariate logistic regression, the odds (OR second year/first year = 1.80; 95% CI, [1.18–2.73], OR third year/first year = 1.53; 95% CI, [1.01–2.32]) of the students of second year and third year were respectively about 80 and 53% higher than students of first year to have correct perception of barriers. While the analysis of association between the perception of beliefs & barriers and the other socio demographic variables including “sexual experience” found that there was no statistically significant relationship.

**Table 2:** The association between the perception of health beliefs & barriers, socio demographic variables and sexual experience of the participants

Variables	The perception of health beliefs & barriers							
Socio demographic	Total ***n*** = 710	Correct perception ***n*** = 540 (%)	OR	95% CI	***P***-value	AOR	95% CI	***P***-value
Age group, year					0.636			
*18–20*	389	288 (74.04%)	Ref					
*21–24*	292	229 (78.42%)	1.25	0.87–1.79				
*25–28*	18	13 (72.22%)	0.57	0.20–1.61				
*28+*	11	10 (90.91%)	1.54	0.33–7.23				
Gender					0.711			
*Male*	309	230 (74.43%)	Ref					
*Female*	401	310 (77.31%)	1.16	0.83–1.65				
Residence					0.088			
*Urban*	469	363 (77.40%)	Ref					
*Rural*	241	177 (73.44%)	0.94	0.66–1.36				
Economic status					0.344			
*High*	43	34 (79.07%)	Ref					
*Middle*	625	469 (75.04%)	1.46	0.74–2.86				
*Low*	42	37 (88.10%)	0.90	0.36–2.23				
Civil status					0.846			
*Single*	589	447 (75.89%)	Ref					
*Coexistent*	63	47 (74.60%)	3.30	1.06–5.95				
*Divorced*	6	4 (66.67%)	2.28	1.06–4.91				
*Married*	52	42 (80.77%)	0.81	0.44–1.49				
Field of study					**0.015**			**0.000**
*Non health sciences*	424	308 (72.64%)	Ref			Ref		
*Health sciences*	286	232 (81.11%)	1.61	1.12–2.32		1.67	1.15–2.41	
Academic year					**0.016**			**0.002**
*1st year*	232	158 (68.10%)	Ref			Ref		
*2nd year*	249	203 (81.53%)	1.80	1.18–2.73		1.88	1.23–2.87	
*3rd year*	229	179 (78.17%)	1.53	1.01–2.32		1.52	1.00–2.31	
Have you had sexual intercourse?					0.151			
*No*	228	173 (75.88%)	Ref					
*Yes*	482	367 (76.14%)	1.39	0.97–1.99				

Referring to *multivariate logistic regression* the socio-demographic factors associated with correct perceptions were: field of study (AOR = 1.67; [1.15–2.41]) and academic year (AOR second year/first year = 1.88; [1.23–2.87], AOR third year/first year = 1.52; 95% CI [1.00–2.31]). These results showed that the students of second and third year were respectively 88 and 52% more likely than first year ones to have correct perceptions. Also, the health science students were 67% more likely than non health sciences ones to have correct perceptions.

Regarding ***the association between students’ knowledge about the preventive methods and the perceived barrier, shown in *** Table 3, the results found that 75% of students (411) who agreed that condom use reduces the risk of HIV infection also believe that using it may seem like an insult to partner. The bivariate analysis with logistic regression between above variables showed that the students with correct answers about knowledge on preventive methods related to HIV infection were 65% less likely to have positive perceived barriers compared to those with incorrect knowledges (OR = 0.35, 95% CI [0.24–0.50]).

**Table 3:** The relationship between student’s knowledge on preventive methods and the perceived barrier

Knowledge on preventive methods: “Using the condom during the sexual act may reduce the risk of HIV infection”	Perceived barrier “Using a condom seems like an insult to my partner”				
	*Correct answer*	*Incorrect answer*	Bivariate analysis		
			Total	OR	95% CI
*Incorrect answer*	79 (11.13%)	81 (11.41%)	160 (22.54%)	Ref	
*Correct answer*	139 (19.57%)	411 (57.89%)	550 (77.46%)	0.35	0.24–0.50
**Total**	218 (30.70%)	492 (69.30%)	710 (100.00%)		

*P*-value: *p* = 0.000 ≤ 0.05

Our study, also analyzed ***the association between VCT uptake and explanatory factors among students with sexual experience***, considered as a subgroup of students with potential risky sexual behaviors. This association is shown in Table 4***.*** According to the association between students’ sexual practices and VCT uptake, the majority of the students with sexual experience (78%) didn’t used VCT services. One hundred six students declared that are tested for HIV, from which 47% females and 53% males. So, there is a difference of 5.67% in favour of males. The results of bivariate logistic regression analysis for the relationship between VCT uptake and socio demographic variables showed that there was a significant statistical association with variables “residence” (*p* = 0.007 < 0.05) and “academic year” (*p* = 0.001 < 0.05). According to residence, 26% of participants from urban areas and 15% from rural areas reported that are tested for HIV. Furthermore, 31% of the students of the second year followed by those of the third year (18%) and the first year (17%) resulted to have testing for HIV. The odds of VCT uptake among students from the rural areas were 2 times lower than those from urban areas (OR = 0.48, 95% CI, [0.29–0.80]). As for academic year, the students of second year were 13% more likely than of first year to have HIV testing (OR = 1.13; 95% CI [0.63–2.04]). The students of third year were 2.29 times more likely than of first year ones to have HIV testing (OR = 2.29; 95% CI, [1.33–3.96]).

**Table 4:** The association between VCT uptake and explanatory factors among students with sexual experience

Variables	Voluntary testing for HIV Did you use VCT service?							
Socio-demographic	Total ***n*** = 482	Yes ***n*** = 106 (%)	OR	95% CI	***P***-value	AOR	95% CI	***P***-value
Age group, year					0.403			
*18–20*	232	54 (23.28%)	Ref					
*21–24*	225	48 (21.33%)	0.89	0.58–1.39				
*25–28*	15	1 (6.67%)	0.24	0.03–1.87				
*28+*	10	3 (30.00%)	1.44	0.36–5.78				
Gender					0.114			
*Male*	290	56 (19.31%)	Ref					
*Female*	192	50 (26.04%)	1.51	0.97–2.33				
Residence					**0.007**			**0.005**
*Urban*	316	82 (25.95%)	Ref			Ref		
*Rural*	166	24 (14.46%)	0.48	0.29–0.80		0.50	0.30–0.82	
Economic status					0.980			
*High*	27	6 (22.22%)	Ref					
*Middle*	426	94 (22.07%)	0.99	0.39–2.53				
*Low*	29	6 (20.69%)	0.91	0.26–3.27				
Civil status					0.280			
*Single*	362	71 (19.61%)	Ref					
*Married*	51	16 (31.37%)	2.11	0.38–11.72				
*Divorced*	6	2 (33.33%)	1.56	0.84–2.87				
*Coexistent*	63	17 (26.98%)	1.93	1.01–3.67				
Field of study					0.459			
*Non health sciences*	329	72 (21.88%)	Ref					
*Health sciences*	153	34 (22.22%)	1.03	0.65–1.64				
Academic year					**0.001**			**0.003**
*1st year*	144	24 (16.67%)	Ref			Ref		
*2nd year*	170	52 (30.59%)	1.13	0.63–2.04		2.31	1.33–3.99	
*3rd year*	168	30 (17.86%)	2.29	1.33–3.96		1.18	0.65–2.13	
The age of first sexual intercourse					0.087			
*Less than 14 years old*	22	4 (18.18%)	Ref					
*15–18 years*	249	54 (21.69%)	1.31	0.43–4.01				
*19 and older*	211	48 (22.75%)	1.37	0.45–4.23				
Number of sexual partners					0.905			
*1 partner*	249	56 (22.49%)	Ref					
*2 partners*	102	23 (22.55%)	0.74	0.40–1.37				
*3 partners or more*	131	27 (20.61%)	0.88	0.54–1.42				

While the analysis of association with the other socio demographic variables found that there was no statistically significant relationship.

The results from bivariate analysis with logistic regression, also showed that there was no significant statistical association between VCT uptake (dependent variable) and independent variables as: age of first sexual intercourse (*p* = 0.874), the number of sexual partners (*p* = 0.905) and sexual experience of participants (*p* = 0.065). So, for each number of declared sexual partners, over 70% of the students did not get tested for HIV.

According to the above analysis, only two variables (residence and academic year) can be entered into *multivariate analysis* using multinomial logistic regression. The findings indicated that the students from the rural area (AOR = 0.50, 95% CI [0.30–0.82]) and those of first academic year (AOR second/first year =2.31, 95% CI [1.33–3.99], AOR third/first year =1.18, 95% CI [0.65–2.13]) were less likely to use HIV testing, which reinforce the results obtained from the bivariate analysis.

On the other hand, there was a significant statistical association between the variables “I would not go to a local clinic to be tested for HIV because then everyone would know my status” and “VCT uptake” (*p* = 0.012) shown in Table 5. Additionally, the findings revealed that 81% of students who had a correct barrier’s perception concluded that would not get tested for HIV. Referring to the bivariate regression analysis the students who had correct perception about barrier to HIV testing were 44% less likely to use VCT service as compared to their counterparts. (OR = 0.56; 95% CI [0.36–0.88]).

**Table 5:** The association between VCT uptake and perceived barrier related to HIV testing

Perceived barrier I would not go to a clinic to get tested for HIV because everyone would know my HIV status	VCT uptake Have you been tested for HIV?			Bivariate analysis	
	*Yes*	*No*	Total	OR	95% CI
	n (%)	n (%)			
*Wrong answer*	43 (8.92%)	104 (21.58%)	147 (30.50%)	Ref	
*Correct answer*	63 (10.07%)	272 (56.43%)	335 (69.50%)	0.56	0.36–0.88
**Total**	106 (21.99%)	376 (78.01%)	482 (100.00%)		

*P*-value: *p* = 0.012 ≤ 0.05

## Discussion

In the present study, the perception of health beliefs and barriers shows a satisfactory result and comparable to other studies [17, 23]. Only 24% of the students have incorrect perceptions. It is very interesting the fact that the higher percentage of the incorrect answers of the participants was found to be related to *the feeling of being insulted* of one partner when the other suggested the use of condom during the sexual act. This feeling is considered in literature as a component of *the perceived barriers* based on Health Beliefs Model related to HIV/AIDS and is *considered to be one of the factors that affect the condom use* [12, 24, 25]. In order to better analyze this factor in our study, we assessed the association between students knowledge about condom use as a preventive method and the barriers that may interfere on its use. There is a significant statistical association between the two above variables (*p* < 0.000) that is consistent with other studies [13, 26] which found that the *perception of barriers was statistically related to the reduction of the condom use among university students.*

In terms of health beliefs related to HIV infection, our study found that participating students were more likely to have sexual intercourse with more than one partner, without being afraid of HIV infection, pretending that they should be worried only when they have serious clinical signs. This finding indicates that a proportion of students are engaged in risky sexual behaviours that may expose them to risk of contracting STD including HIV infection. Interestingly, a significant proportion of students believe the misconception regarding HIV diagnosis, including that having an HIV test within 1 week of risk behaviour will tell if a person has HIV. These findings are reflected in similar other studies [27] that also show limited knowledge on this issue, which raises the need to have informing and preventing ongoing activities. About a third of the students believed that those who contracted HIV through illegal behaviour should not be treated in hospital and clinics. This may indicate that negative beliefs can contribute to continued stigma towards key at-risk populations and may be a barrier to HIV prevention efforts.

In relation to socio-demographic factors, we observed a significant correlation between health beliefs & barriers and academic year, which is similar to international studies. These findings showed that the students of second and third year were more likely to have correct perceptions that is probably because the university curriculum structure teaches HIV topic in the 2nd year and beyond [20, 27]. While it is encouraging to note that health science students and those with sexual experiences have more correct beliefs and attitudes related to HIV, which is consistent with previous studies [20, 27, 28].

In addition, the incorrect perception found to have the single students followed by married ones. These results are consistent with those of Sarcar, which found out that the socio-cultural aspects and the beliefs regarding the sexual act serve as barriers or influence on the condom use in marriage relations or the married students in India, while in other countries as Brazil, Africa, Mexico etc. the fear of condom use shows the absence of faith within the couple, and acts as a barrier of not using the condom from these couples [26] .

**Counseling** and **testing** for HIV/AIDS is another valued literature method which plays an important role in screening and keeping this infection under control. Our study analyzed this role and found out a low prevalence of voluntary testing for HIV among the students with sexual experience compared to other similar studies [18, 29]. In relation to demographic factors we notice that there is a slight change in males who have the tendency to use VCT services more than females, which can be explained by the fact that albanian females hesitate to get tested for HIV due to the prejudice [30]. Contrary, other studies revealed a higher incidence of VCT uptake in females compared to males [31–34], probably because HIV effects last much longer on females and they are aware of this fact.

Indeed, HIV infection is equally dangerous for males and females, which means that the VCT uptake has the same level of importance for both genders. Meanwhile our study shows *a statistically significant correlation between VCT practice and the residence* of the participants with sexual experience. The students who live in the city have a higher probability to use VCT services than the ones who live in the rural areas. Referring the data from Universal Periodic Review of Albania 2018, the people who live in rural areas and the ones with lower income do not refer to the primary health care, because going to health care institutions of a higher level asks for more money and time [35]. This is why the students who show a higher percentage of using VCT services are the ones that live in urban areas. The association *between VCT practices and sexual behaviours shows that there is no statistical association*, which means that the number of sexual partners and the age of first sexual intercourse do not have an impact on the fact that the students use VCT services or not. These results are in contrary to another study which showed that participants engaging in higher-risk sexual behaviours were more likely to be tested for HIV [36]. The results show that regardless the students’ exposure to risky behaviours, there is a low number of voluntary testing for HIV even within the risky groups, similar to other studies previously carried out in Albania [35, 37]. Additionally, Albania is a traditional and patriarchal society in which stigma and discrimination play a significant role in preventing sexually active young people like tertiary level students accessing HIV testing for earlier diagnosis and treatment of HIV infection [30]. Regarding the fact that the barriers may have a negative impact on VCT practice, our study found that *the fear of stigmatization* may be one of them, which is similar to other studies [9, 17, 30, 32, 38]. As a whole, our results demonstrate that the students’beliefs may be barriers to universal access to use VCT services.

### Limitations

There were several limitations to the study. The first one was related to the self-reported answers, which means that the results are affected to an over or under reporting. Moreover, the subject of the study consists of a delicate issue such as sexual behaviour. The second one is related to the development of the questionaire with closed questions, that prevents us from obtaining a more detailed information from the participants. Finally, the findings cannot be generalized to all university students in Albania because the sample was conveniently collected from one university in one city. Despite all of these limitations, we believe this study might be a reasonable source of information for researchers and policymakers.

## Conclusions

The results of this study showed that the perceptions of barriers are good predictors of the preventive behaviours toward HIV infection. Nevertheless, this study discovered that “the feeling of insult” related to condom use and “the fear of personal consequence” related to HIV testing such as “fear of losing partner”, were the main barriers that influence health services uptake, answering the research question of the study. In addition, the prevalence of VCT services resulted low and the fear of stigmatization was considered another potential barrier to non-use of them by students. The present study provides evidence to suggest that creating awareness about prevention and screening methods for HIV among student community, especially those from non-health sciences, could contribute to increased uptake of the VCT services, the condom use and to reduce the identified stigmatizing barriers. Identifying the barriers related to preventive and screening methods for HIV would help the health practitioners to implement better professional education, promote healthy sexual behaviors and increase access to quality services. This paper highlights the need to integrate stigma reduction with HIV prevention activities and the importance of investigating the impacts of the healthcare utilization among young people especially students with high-risk behaviors and those from rural areas which will constitute formidable challenges to the international community.

## Data Availability

The datasets used and/or analysed during the current study are available from the corresponding author on reasonable request.

## Abbreviations

HIV: Human Immunodeficiency Virus
AIDS: Acquired Immunodeficiency Syndrome
VCT: Voluntary Counseling and Testing
ART: Antiretroviral Therapy
WHO: World Health Organization
ECDC: European Centre for Disease Prevention and Control
STD: Sexually Transmitted Diseases
KAPB: Knowledge, Attitude, Practice and Belief survey
SAS: Statistical Analysis System
CI: Confidence of Interval
AOR: Adjusted Odds Ratio
